# The perceived value and impact of virtual simulation-based education on students’ learning: a mixed methods study

**DOI:** 10.1186/s12909-022-03912-8

**Published:** 2022-11-30

**Authors:** Amanda K. Edgar, Susie Macfarlane, Elissa J. Kiddell, James A. Armitage, Ryan J. Wood-Bradley

**Affiliations:** 1 School of Medicine (Optometry), Deakin University, 75 Pigdons Road, Waurn Ponds, 3216 Australia; 2 Deakin Learning Futures, Deakin University, Burwood, VIC 3125 Australia

**Keywords:** Virtual simulation, Optometric education, Clinical reasoning, Assessment

## Abstract

**Background:**

Virtual simulations are used throughout healthcare training programs to enable development of clinical skills, however the potential for virtual simulation to enhance cognitive and affective skills is less well understood. This study explored pre-clinical optometry students’ perceptions of the impact of virtual simulation on the development of core competency skills including patient-centred care, communication, scientific literacy, and evidence-based practice.

**Methods:**

A mixed methods study was conducted using pre-existing anonymized data from an electronic survey distributed to pre-clinical optometry students enrolled in the double degree Bachelor of Vision Science/Master of Optometry at Deakin University, Australia. The data were interpreted using descriptive statistics and qualitative analysis using constant comparison for thematic analysis.

**Results:**

A total of 51 responses were analyzed. Students reported that virtual simulation motivated them to become an optometrist (93%) and to learn beyond the course material (77%). Students reported that after participating in the virtual simulation, their core competency skills improved: patient-centered care (100%) evidence-based practice (93%) and clinical reasoning (93%). The themes identified through qualitative analysis were: enablers to cognitive experience in virtual simulation in optometry education, realism of the virtual simulation design, dimensions of fidelity in virtual simulations design replicated the complexity of the optometric environment, virtual simulation as an enabler for learning and assessment in optometry education, a place to develop cognitive and affective skills and application of learning in the virtual simulation developed an appreciation of future roles and professional identity.

**Conclusion:**

Optometry students perceived virtual simulation in optometric education as a valuable training and assessment strategy enabled by qualities that generate contextual, cognitive, functional, task and psychological fidelity. The data provide insight to inform how optometry educators can incorporate simulation into the curriculum.

**Supplementary Information:**

The online version contains supplementary material available at 10.1186/s12909-022-03912-8.

## Background

It *“felt very real.”* (S7)*.* Virtual simulation has long been accepted as an assessment strategy in healthcare education [1, 2]. For this study, virtual simulation is defined as a partially immersive, screen-based experience where a simulation injects the human in a role where they perform motor control skills, decision skills and/or communication skills [3]. Virtual simulation has successfully provided education and assessment activities across various healthcare disciplines to facilitate the development and refinement of clinical skills and work routines [4–8]. There is also evidence that virtual simulation can be used to assist healthcare trainees in the pre-clinical phase of their education to develop cognitive skills such as patient-centered care and evidence-based practice, but in optometry this evidence is more limited than for clinical procedural skill development [9]. To be judged as a competent practitioner and demonstrate entry level competence, optometry students need to develop clinical and affective skills such as the clinical reasoning, interpersonal skills, cultural safety, evidence-based practice, and patient-centered care described in entry level standards [10]. The use of virtual simulation and its role in developing these skills in optometry education is yet to be established.

Virtual simulation allows customization of clinical learning experiences that are directly related to a professional setting and patient population. This learning strategy has become a key feature in healthcare programs that aim to prepare students for their future workplace and professional roles, with evidence showing that virtual simulations can help medical and nursing students, as well as healthcare professionals, develop crucial skills required for clinical practice [11–15]. With these advantages, the inclusion of realistic virtual simulated experiences to produce work-ready and work-safe graduates has become a resource used in healthcare education. Therefore, this study aimed to answer the question whether students perceived the use of virtual simulation in optometry education as a method of offering additional learning and assessment strategies that foster the development and refinement of core competency skills such as cognitive and affective skills.

Affective and cognitive skills are a component of the core competencies that are essential for qualification as an entry level optometrist [10]. These are the skills that allow an optometric clinician to associate knowledge with evidence-based research and apply it through decision making and clinical judgement [16]. There has been some preliminary investigation of the use of virtual simulation in optometric education - for example to improve clinical procedural skills such as refraction and binocular indirect ophthalmoscopy, and to develop anatomical knowledge with anatomy simulators [17–20]. However, there have been no published investigations of the affordances of virtual simulation in optometric education to develop students’ cognitive and affective skills.

As educators in an optometry program, our observation of students in pre-clinical years is that they struggle at times to see the connection between what they are learning in the pre-clinical phase and their future roles as a clinician. To strengthen the connection between pre-clinical skill development and the application of these skills in clinical practice, we developed a teaching environment that would bring to life this nexus in a virtual simulation. Virtual simulation was selected as it has been shown to be effective in other disciplines at motivating students by supporting them to identify the subject matter being delivered in learning and assessment activities as relevant to their future roles as practitioners [21]. A virtual simulated assessment task was designed for first year students enrolled in the Bachelor of Vision Science/Master of Optometry program in the Deakin University School of Medicine. A case-based approach was chosen due to the strong body of evidence that it is an effective intervention for building clinical competency [22]. This task was carefully scaffolded to integrate and contextualise the evidence-based practice and patient-centred care learning opportunities and included guided problem-centred instruction from clinical experts so that the approach was suitable for first year students with low levels of discipline-specific knowledge [23]. Clinical experts also provided an active learning environment by framing and debriefing students’ experience of the virtual simulation and allowing them to reflect on their performance [24].

The effectiveness of virtual simulations in supporting students’ engagement and learning depends on the extent to which learners perceive it to be valuable and easy to use [24]. This study aimed to investigate pre-clinical optometry students’ level of acceptance of virtual simulation for learning and the perceived effect on the development of cognitive and affective skills as core professional competency skills.

The following research questions form the basis of the study: 1) what are the barriers to and enablers of optometry students accepting virtual simulated learning and assessment strategies in optometry education; 2) do virtual simulation simulated learning and assessment strategies motivate optometry students to study through an enhanced connection to practice; 3) what is the affordance of the Virtual Deakin Collaborative Eye Care Clinic (VDCECC) as a learning environment for students to begin learning cognitive and affective core competency skills such as clinical reasoning, evidence-based practice and communication skills; 4) how do virtual simulated learning and assessment strategies influence optometry students’ perceived development of professional capabilities; and 5) what is the extent to which optometry students perceive the value of virtual simulated learning and assessment strategies?

## Methods

### Design

This study was designed to assess the perceived value of virtual simulation-based learning and assessment strategies for education in pre-clinical optometry education. This study uses pre-existing anonymized data and was judged by the Deakin University Faculty of Health Ethics Review Committee as being exempt from requiring approval for use or the need for consent considering the survey data reported was collected as part of quality assurance evaluation of teaching activities and all responses were submitted anonymously and voluntarily.

### Setting

This study was conducted at Deakin University School of Medicine, Faculty of Health in the optometry course using pre-existing data collected from quality assurance activities within the first year of the curriculum of the Bachelor of Vision Science/Master of Optometry course. This course is a ten-trimester double degree over three and a half years. Of the approximately 80 first year students, the majority are school leavers with no previous clinical work experience. The first year of the curriculum is pre-clinical and focuses on basic science and foundational skills required for clinical practice. In subsequent year levels the curriculum is designed around problem-based learning, clinical skills development and clinical placements. The unit of study for which this evaluation was conducted was an 11 week first-year unit designed to teach foundational skills such as evidence-based practice, patient-centred care, and shared-decision making. Student perceptions were collected at the completion of the 11th week using an anonymous and voluntary survey instrument as part of a quality assurance evaluation of this novel teaching activity.

#### Virtual simulation platform

**Fig. 1:**
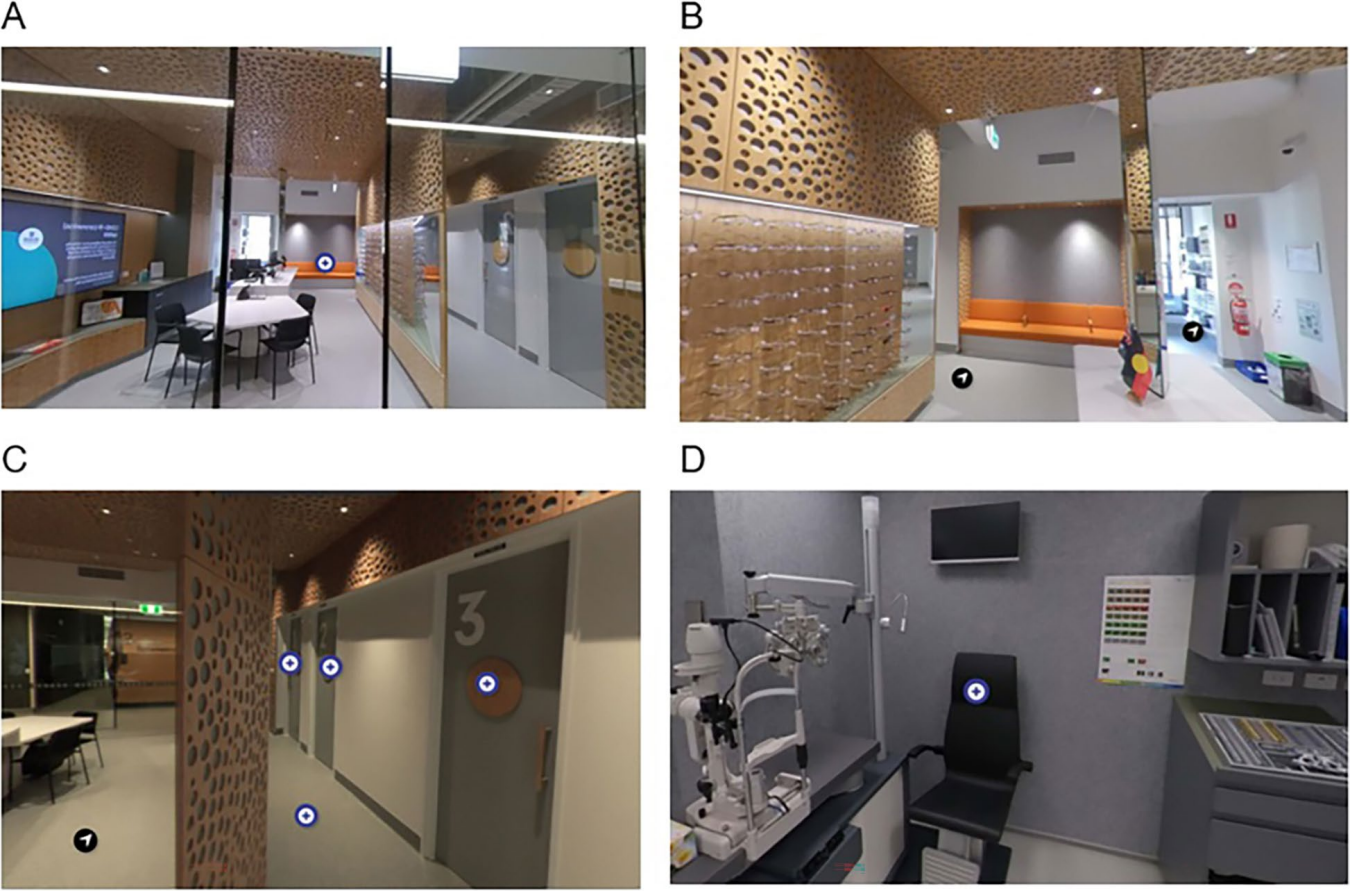
Screen shots from the user view of the VDCECC. **A** Users view from their personal device of the entry to the VDCECC replicates the physical entry to the university clinic. **B** Users view moving through the lobby of the VDCECC where they can see spectacles for sale and patient waiting area. **C** Users view showing the options to click on the doors and enter different consultation rooms or move through the hallways of the clinic. **D** Users view of the consultation rooms that contain optometric equipment required to perform clinical testing. Abbreviations: VDCECC; Virtual Deakin Collaborative Eye Care Clinic

The learning activities were conducted within a virtual simulation of the Deakin Collaborative Eye Care Clinic (DCECC). DCECC was chosen due to its relevance to clinical practice and optometry students’ roles as it is a university optometry clinic with direct patient care and clinical research activities. It also provides senior year students the opportunity to participate in student-led clinics and undertake competency-based clinical assessments such as Integrated Performance Assessments and Objective Structured Clinical Examinations. Based on its future role in students’ academic experience and the clear relevance to their future professional practice, this location was chosen to be simulated as a Virtual Deakin Collaborative Eye Care Clinic (VDCECC) to support fidelity of the virtual simulation. Interactive media in the form of 360-degree photography was used to recreate the clinic environment. A clinician (AE) designed and developed the virtual 360-degree environment using embedded in H5P, an HTML5-based interactive software (HTML5 Plugin H5P, see Fig. 1) and this was presented to students on the University learning management system (Cloud Deakin, D2L, Brightspace). To reduce biases an academic who was not a healthcare clinician and delivers the teaching material (RWB) reviewed the virtual simulation to ensure it mirrored the physical space and could be used by 1st year optometry students. The result was a web-based platform for virtual simulation that can be accessed via personal computer, laptop, tablet, or mobile phone anywhere with internet connection and was used by students enrolled in this unit of study to complete assessment tasks.

#### The assessment

The assessment was a virtual simulation activity where a group of 6-7 students worked together over the trimester to perform an evidence-based eye examination demonstrating patient centred care with a virtual patient in the VDCECC. This task would be beyond the ability of these pre-clinical students as they were yet to be taught the elements of an eye examination. It was therefore essential to scaffold the assessment task to spark situational interest while also managing the level of complexity to enable learners to focus on the intended learning outcomes [25, 26]. This was done by emphasising that students were not assessed on the basis of diagnosis of or proposing a management strategy for patients (optometric-specific knowledge), but rather robustness of the evidence-based practice and patient-centred care decisions that aligned with their learning in the academic unit of study. Each week, the simulation replicated a different stage of an eye examination; at the beginning of the trimester the students were provided the introductory details of their virtual patient and by week 10 finalised the examination with management of a particular diagnosis. This learning strategy followed a recommend three phase assembly of framing, virtual simulation and debriefing [27, 28].

**Fig. 2:**
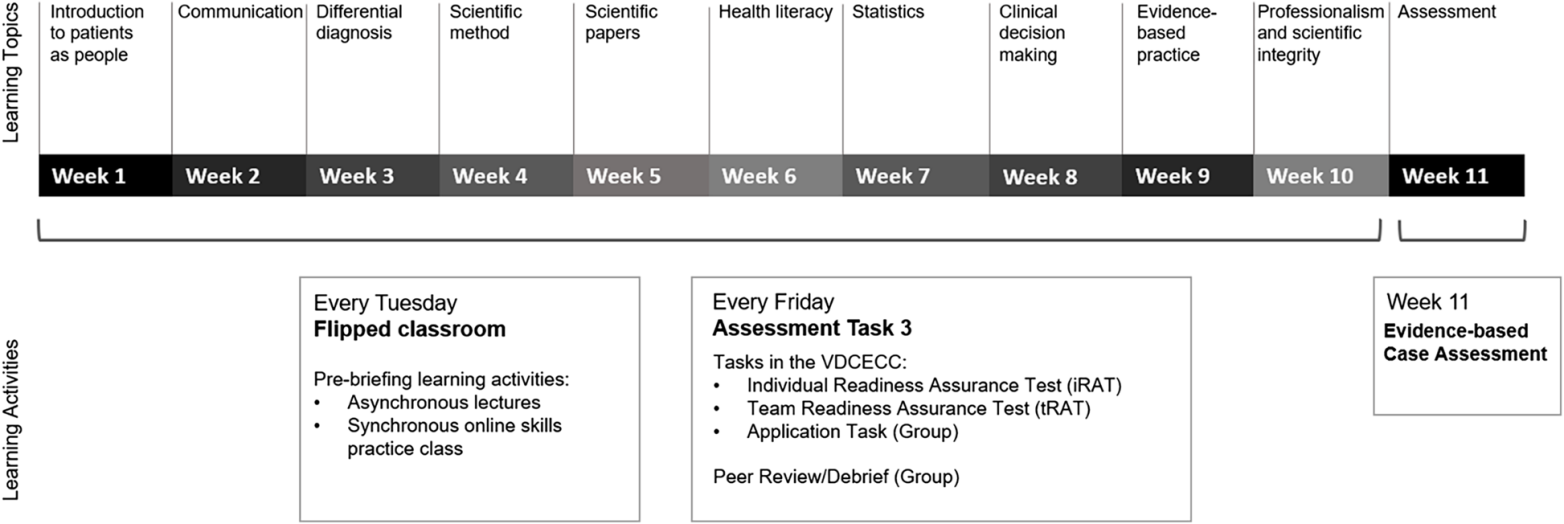
Structure of the 11-week trimester learning activities and examples of the topics that were covered within the teaching unit for the first 10 weeks and a final case-based assessment in week 11. Abbreviations: iRAT, Individual readiness assessment task; tRAT, team-based readiness assessment task; AppEx, Application Task

The unit structure allowed for a topic to be delivered each week of the 11-week trimester (see Fig. 2) and for theory to be applied in the assessment task. At the start of each week, pre-briefing or framing material was provided in the form of pre-recorded lectures that delivered theory relevant to the assessment and an online live class was held to help students practice applying theory from the weekly learning modules. This provided students both synchronous and asynchronous opportunities to engage with learning materials. Feedback and discussion in these classes were facilitated by an academic and a clinical optometrist. At the end of the week, each group completed the summative assessment over 10 weeks, with a final assessment in week 11.

In the assessment task, students entered the VDCECC to apply skills they had learnt during the week on their virtual patient. Following the virtual simulation, students debriefed with peers, academics and clinicians to extend their developing professional identity and connect them further to the professional world outside of the university [26]. Based on their weekly assessment submission, students received further information on their virtual patient in the subsequent week.

The assessment task was carefully pitched to a first-year student’s level to introduce them to the unit content in the context of a common optometry consultation in Australia with a patient who had diabetes. The virtual simulation was low fidelity for this early stage of learning to reduce cognitive load from extraneous information, based on evidence there is a limit to the amount of information people can process simultaneously [29]. To construct the assessment task, two clinical optometrists and educators identified the key components of the role of an optometrist during recommended procedures for examining patients. The decision was made to base the procedure on examining a patient with diabetes as this afforded the ability to introduce the breadth of optometric techniques and management options as a snapshot of a professional consult. Students also chose a ‘puzzle piece’, symbolising a clinical choice relating to their virtual patient, that acted as a cue to the student to move through the clinical scenario to reach the intended learning outcomes. The clinical choice provided students the opportunity to practice a range of clinical competencies such as communication by selecting a question to ask the patient, clinical reasoning by choosing a test to perform, or diagnostic decision making by selecting a preferred differential diagnosis. Based upon the puzzle piece chosen, the group would receive further clinical information unique to their virtual patient the following week such as a response to the question they chose to ask or clinical test results from a test they selected to perform. This cycle of completing the weekly content, engaging with the live online classes, completing the assessment, and choosing a puzzle piece with information about the virtual patient that prompted the practice of their clinical competency skills, continued for 10 weeks. At the end of 10 weeks each group had a unique virtual patient experience based on their clinical reasoning but received the same teaching material to apply to the assessment task.

### Instrument

A 21-item survey instrument (see Additional file 1) was developed by the teaching team to evaluate students’ perceptions of their experience in the virtual simulation as part of a quality assurance process of a novel teaching environment as no published instruments were available. The researchers determined content and construct validity from reviewing the questions and their concepts using their experience; 3 experienced optometric educators, 1 an anatomist and 2 qualified optometrists as well as 2 instructional design experts. Students rated the value of the VDCECC on authenticity, realism, the preference for virtual simulation in optometric education and the improvement of their clinical skills through virtual simulation in the VDCECC. Students were asked to rank on a five-point Likert scale how strongly they agreed with a statement from 1 (strongly disagree) to 5 (strongly agree). Open ended questions invited students to discuss their experiences in relation to their answers and how beneficial they perceived the virtual simulation was for optometry education.

An online software platform (Qualtrics, Provo, UT) was used to distribute the evaluation survey as a link posted to the announcement board on the unit learning management system as an optional and anonymous activity that was not assessed and was available to be accessed by all students enrolled in the unit. The survey completion required approximately 15 minutes. This evaluation process was undertaken to evaluate the student experience of the first implementation of this teaching and assessment strategy and platform within the program, and as such the instrument was not formally validated.

### Data analysis

Quantitative data were obtained from the 5-point rating scales. The responses were analysed using raw numbers, frequencies, percentage of agreement and disagreement and descriptive statistics where appropriate using Statistical Package for Social Sciences (SPSS) (version 21). Answers to questions 11.1-11.4, 14-15 and 17 are displayed in Tables 1 and 2 below in percentage for each question.

**Table 1:** Student perception of the virtual simulation in the Virtual Deakin Collaborative Eyecare Clinic

Question:	Strongly Disagree n (%)	Disagree n (%)	Unsure	Agree	Strongly Agree
*The virtual simulation was realistic*	0%	2 (7%)	4 (10%)	14 (48%)	10 (35%)
*The virtual simulation was relevant to my learning*	0%	0%	2 (7%)	14 (48%)	14 (48%)
*The virtual simulation motivated me to learn the unit’s content*	0%	2 (7%)	2 (7%)	14 (48%)	12 (38%)
*The virtual simulation motivated me to research topics beyond the material provided*	0%	3 (10%)	4(14%)	15(50%)	8(28%)
*After participating in the virtual simulation… I have a good understanding of how I will behave as an optometrist*	0%	0%	4(14%)	17(63%)	6(23%)
*After participating in the virtual simulation… the next time I encounter a real or simulated patient I will feel more confident*	0%	0%	5(18%)	11(41%)	11(41%)

**Table 2:** Students’ perception of core clinical skill development through the virtual simulation

Core clinical skill	Reduced a great deal	Reduced	Unsure	Improved	Improved a great deal
Patient-centred care	0%	0%	0%	8 (25%)	21 (75%)
Evidence based practice	0%	0%	2 (7%)	8 (25%)	19 (68%)
Clinical Reasoning	0%	0%	2 (7%)	10 (32%)	17 (61%)
Clinical Knowledge	0%	0%	2 (7%)	14 (48%)	14 (48%)
Communication skills	0%	0%	4 (14%)	12 (38%)	14 (48%)

Thematic analysis was performed on text responses to open ended questions by two independent coders (AE and RWB) on three students’ surveys for thematic content, and these coders developed a draft set of themes. Comments were initially reviewed and mapped to thematic domains with co-authors (SM and JK). AE and RWB then coded 10% of responses and discrepancies in major themes were reviewed with co-authors to ensure there was alignment of the codes with the research aims and theoretical approach (SM and JK). AE and RWB independently coded the remaining data using the coding structure and comparison to identify new concepts or categories constantly [30]. This was then reviewed with co-authors (SM and JK) to explore relationships between categories and discussing areas of overlapping themes, disagreement, merger of codes and exclusion of content until consensus was reached to conceptualize the data [30]. Responses were then coded by one independent coder (JA) and representative comments by students on major themes were chosen and agreed upon by all co-authors and then extracted for illustration of themes. Sample size was based on convenience sample of all students enrolled in the unit - those that filled in the initial quality assurance and unit review survey.

## Results

Of 80 students that were given the option to complete the online voluntary and anonymous evaluation survey, 51 responded (64%) with a mean age of 20 (std 3.24 years, 18-33) and all responses were included in the analysis. Given the high response rate these responses are representative of the cohort. These first-year students who completed the evaluation had diverse exposures to optometry: 43 (83%) had visited an optometry practice for retail spectacle, contact lens or sunglass purchase, 39 (76%) had received eye care a patient in an optometry practice, 11 (22%) students had worked in an optometry practice and 5 (10%) had visited a university clinic. The majority of students were high school leavers (*n* = 32, 63%) while a quarter had completed a bachelor’s degree level qualification (*n* = 13, 25%). No students had studied in any other university optometry program.

The findings from thematic analysis are presented alongside the quantitative results in Tables 1 and 2 to support triangulation of qualitative insights. There were six themes and their subthemes identified from an initial 31 codes once data saturation was achieved. Quotes are reported verbatim from students in italics with clarifying insertions in square brackets and an identification number. The quantitative results are presented as a percentage of the responses.

### Theme 1: enablers to cognitive experience in virtual simulation in optometry education

Students’ responses indicated that their ability to accept the virtual simulation experience as authentic was dependent on previous exposure to the context of optometry. One student said:

*“I haven’t personally visited an optometry clinic yet, so I am unsure if the virtual clinic is representative or not.”* (S7).

Others commented on how real-life familiarity with optometry practice supported their interpretation of the environment.

*“It looks pretty similar to my experience with eye clinics, with all the equipment there.”* (S2).

### Theme 2: realism of the virtual simulation design

Students described the virtual simulation as operationally realistic enough to feel like work experience and that they could enter the clinic, describing a sense of immersion.

*“I felt like I am in the real clinic and that enhanced my interest.”* (S19).

*“…despite being online it felt very real.”* (S7).

This theme is further supported by quantitative responses suggesting that the level of detail in the VDCECC created an experience that represents a particular world with 80% of students agreed or strongly agreed that the virtual simulation was realistic (Table 1).

The realism of this virtual world allowed the students to feel as though they are involved in metaphysical events that really take place.

“…an indirect experience in the physical environment.” (S41).

The fact that the virtual clinic has properties similar to the working world gave students a sense of immersion and the feeling that they are working as a practitioner. Students said that they.

*“… learn more about the process of seeing a patient in general.”* (S12).

*“… know how it feels to work with patients (and) trying to diagnose them”* (S7).

All students who had previously visited a university optometry clinic agreed that the virtual clinic was an accurate representation of an optometry clinic, 88% of students with retail experience in an optometry practice, 86% who had undertaken an eye test, and 66% who had worked at an optometry practice.

### Theme 3: dimensions of fidelity in virtual simulations design replicated the complexity of the optometric environment

Fidelity is defined as the degree that a simulated experience approaches reality, the believability of the users’ experience (International Association for Clinical Simulation and Learning) [31]. The experience of a virtual simulation is constructed by multiple dimensions of fidelity. During the qualitative analysis of this study researchers identified in student responses the dimensions of fidelity that contributed to their experience of the virtual simulation that approximated optometry practice [31]. The four key dimensions of fidelity that supported the realism of a virtual world in a metaphysical space were coded as the subthemes context, conceptual, functional and task fidelity. One example of context fidelity is illustrated in responses describing features of the physical environment that represent an optometry clinic, such as Student 7’s comment:

*“It represents my idea of a clinic with the patient rooms, front desk, and displays of glasses.”* (S7).

Students also experienced the replicated environment as a space in which activities that occur at an optometry practice could be performed, indicating functional fidelity was an important contribution to the realism of the simulation for some students.

*“… an area for frames to be selected, just like most eye clinics...”* (S41).

*“… found that using this virtual eye clinic gave students a realistic perception of what it’d be like if we were to perform these tasks in real life.”* (S30).

*“Felt like I am in a real clinic.”* (S18).

Task fidelity was also identified by students as an important aspect of the VDCECC learning environment and assessment strategy that enhanced the realism of the simulation through replicating the professional role [32, 33].

*“… using this virtual eye clinic gave students a realistic perception of what it’d be like if we were to perform these tasks in real life.”* (S5)

The realism of the VDCECC environment and the integrated learning and assessment activities relevant to the unit content produced a coherent experience leading to conceptual fidelity [34].

*“The simulation gave us more direction and a sense of importance into what was being taught in that week.”* (S4)

### Theme 4: virtual simulation as an enabler for learning and assessment in optometry education

Exploring the role of virtual simulation researchers found it to be an enabler for learning and assessment.

*“In general, I enjoyed how we got to put the content to use in a practical situation, which further developed my knowledge.”* (S33)

*“… provided greater motivation as the learning felt more engaged than rote learning facts.”* (S14)

In comparison to other learning activities the unit employed the virtual simulation promoted active learning for the students.

*“The virtual simulation is different because it’s not just someone talking at me for an hour. It forces me to actively engage with content.”* (S5)

*“*… *it allows a different perspective where responses can be more considered and less rushed than with an in-person setting.”* (S44)

There was evidence that students perceived the value of the virtual simulation as a learning and assessment environment that allowed safe experimentation.

*“… think it was a good way to experiment and test the waters without much consequence for any mistakes. It gave [students] more confidence to see how different things would work out…”* (S25)

The quantitative responses also demonstrate students’ perception of the virtual simulation as enabled learning through offering another form of teaching and assessment that is relevant and motivated them to seek out more learning. In Table 1 most students agreed or strongly agreed that the virtual simulation was relevant (93%), and increased their motivation to learn unit content (87%) and to research material beyond that provided (77%). After visiting the VDCECC 83% would prefer virtual simulation over a regular lecture in their future studies (Table 1). In terms of their motivation for learning 86% agreed with the statement that the virtual simulated training and learning strategy delivered in the unit motivated them to learn the unit’s content and 76% agreed that it motivated them to learn beyond the material provided in lectures and other teaching activities in the unit*.* Most students (93%) felt that the virtual simulation increased their motivation to become an optometrist.

### Theme 5: a place to develop cognitive and affective skills

In the quantitative data students strongly rated the VDCECC as a place to develop core competency skills for becoming an optometrist as shown in Table 2. They appreciated that the simulation was scaffolded to the appropriate level to begin learning these skills.

Qualitative analysis of open responses gave further understanding that the virtual simulation itself was regarded as a place to develop core conative and affective skills.

*“To me, virtual simulation is simply a good way to introduce first year students to the clinical reasoning that will be expected of them in the future.”* (S14)

When asked what they learnt through the virtual simulation experience, students identified that the virtual simulation experience developed their research skills, clinical reasoning as well as skills involved in interacting with patients.

*“I began developing patient communication skills and further developed my research and collaboration skills.”* (S44)

*“Recognising and diagnosing issues using different diagnoses and other methods.”* (S31)

There were also reflections indicating the simulation supported students to develop their mindset of patient-centred care.

*“Having to consider the patient when helping and communicating to them.”* (S43)

*“It will greatly improve the way we communicate with patients and positively affect our ability to make decision*s.” (S37)

All students expressed that their preference to learn using the virtual simulation in the future. Compared with traditional teaching and learning activities 40% of students preferred virtual simulation, 43% appreciated both modalities, 13% were unsure and 4% preferred traditional learning activities.

### Theme 6: application of learning in the virtual simulation developed an appreciation of future roles and professional identity

Students embraced the opportunity to simulate being a professional in the virtual simulation and enact the role of an optometrist by applying the learning from the unit.

*“I put myself in the shoes of an actual Optometrist.”* (S30)

*“Having to review the information given by the patient and create a list of the differential diagnoses was very helpful to apply my knowledge to an actual clinical scenario, as well subsequently evaluating the results of the eye examination.”* (S34)

Students indicated that the virtual simulation promoted the development of their professional identity.

*“It assisted [first year students] to understand the challenges behind the profession and there are more things than just diagnos[ing] pathology.*” (S25)

*“*… *a hint of how it feels like to work in a real world with patients.”* (S15)

*“… an opportunity to examine the principles behind optometry practice and get the sense of being an optometrist.”* (S28)

*“helped [students] understand that the role of an optometrist extends beyond just caring for the patient’s vision - it involves making sure their overall health and wellbeing is good and properly managed.”* (S29)

In fact, the majority of students reported a positive effect on their professional identify after participating in the virtual simulation, as shown in Table 2. Participation in the simulation increased their confidence for when they next encounter a patient (86%) and their understanding of how they will behave as an optometrist (82%).

## Discussion

This virtual simulation strategy was developed considering pedagogical principles to inform students’ engagement in simulated learning activities. The teaching team designed the VDCECC to enable activities to be aligned to the level of the student and three phases were incorporated into the simulation activity: framing, virtual simulation followed by debriefing [27, 28]. To consider how virtual simulation can be used in optometric education, the enablers for this learning strategy were investigated from the perception of the optometry students. Thematic analysis identified that five domains of fidelity - context, conceptual, functional, task and psychological fidelity - as well as previous experience in an optometric setting, influence students’ perception of the realism of the virtual simulation [30, 34–36].

One aim of this virtual simulated clinical environment was to create a place for teaching and learning activities that links students’ learning to the professional world beyond the university. Students accepted that the VDCECC was a sufficiently authentic replication of an optometry clinic as based on their previous experience in the real-life optometry setting. Given that the University clinic differs from community optometry clinics in a number of areas such as the number of consultation rooms and resources, it is perhaps surprising that most students (74%) agreed the VDCECC was an accurate representation of an optometry clinic. A previous exposure to real-life optometry was an enabler to the cognitive experience in virtual simulation in optometry education. This identifies a previous understanding of the environment or context of a virtual simulation activity for optometry education could support acceptance of the virtual simulated learning strategy.

### Realism

**Fig. 3:**
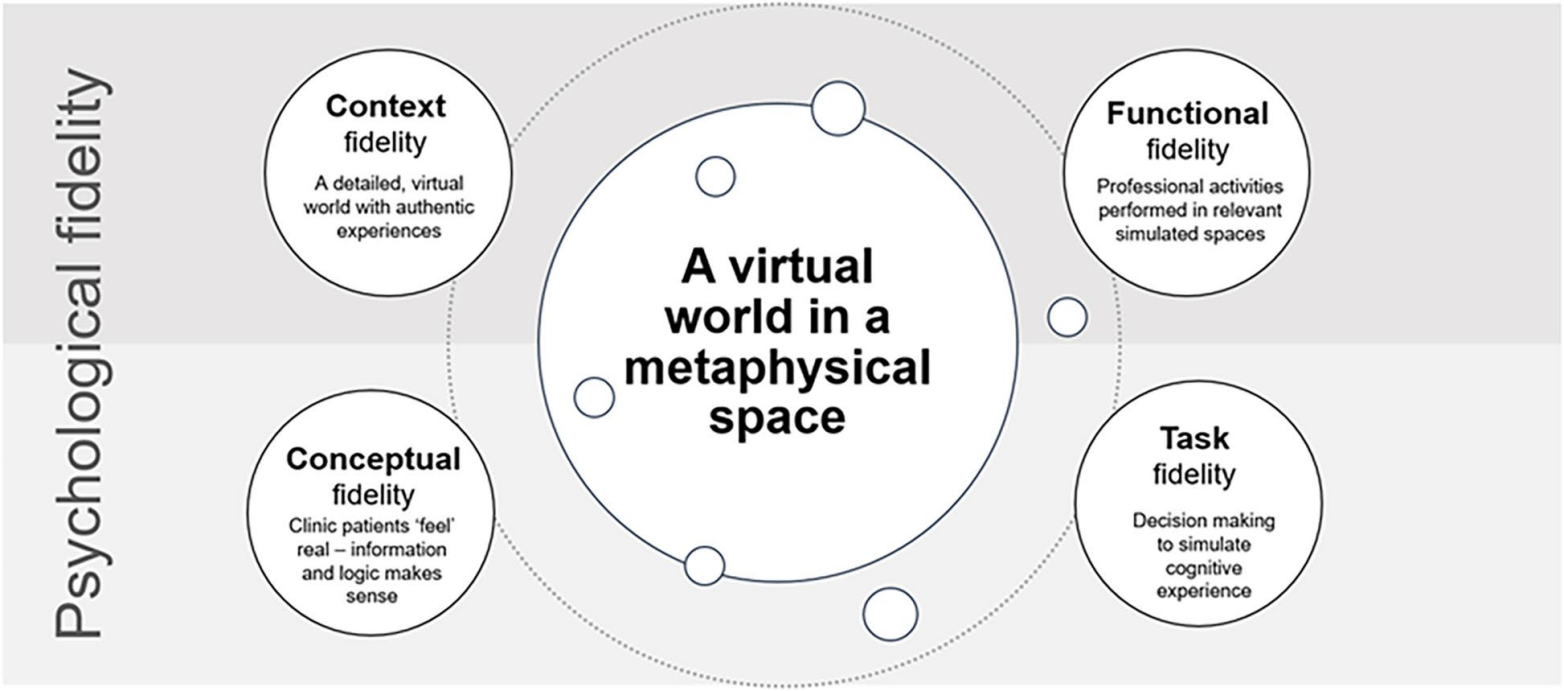
A diagrammatic outline of the sub-themes of fidelity that created layers to build the complex virtual world of optometry in a metaphysical space

The development of skills in virtual simulation has been discussed by literature in context of realism/congruence [32]. Learning is positively affected in simulations that have physical and performance features that match real life experience [37]. The level of detail in the VDCECC, the fidelity (Fig. 3), created the experience for students that the space they occupied represents a particular world, a virtual optometric world. In this virtual world the students identified that the tasks they completed put their learning into context and positioned them as clinicians. In this educational experience, though the content is theoretical, the virtual simulation puts practice theory into reality or virtual reality, with the sense of immersion reported by students creating a real metaphysical place. With the assessment as a virtual simulation activity embedded into the VDCECC replicating real-life scenarios, the experience in this virtual world validated students’ prior experience and reflected the roles they will play in future practice and clinical contexts.

### Fidelity

Fidelity, in the context of learning activities, is an important design feature in developing skills through virtual simulation. This is because the replication of the key features of an activity or learning experience enhances the reality of a simulation [38]. The thematic analysis revealed the VDCECC simulates the complexity of the clinical optometry environment, the professional role of an optometrist and the process of an eye examination rather than the clinical technique or a particular clinical procedure.

The thematic analysis found the following fidelity dimensions were identified by students as supporting the cognitive experience:

- **Context fidelity**: the VDCECC provided sufficient detail of sensory information in the environment and equipment to contribute realistic context to the virtual simulation
- **Functional fidelity**: the VDCECC simulated the physical characteristics of the clinic environment in which an optometrist performs a range of tasks
- **Conceptual fidelity**: the VDCECC generated an experience of conceptual coherence for students through the learning and assessment tasks and problems
- **Task fidelity:** the VDCECC provided appropriate sensory and behavioural features to support the delivery of a dynamic learning environment centred around clinical decision making for a virtual patient.

The qualitative analysis found subthemes demonstrated that the students noticed these dimensions of fidelity helped them draw parallel between what they were experiencing in the virtual simulation and what they expected to be doing as an optometrist, and researchers found this supported an overarching psychological fidelity: that the virtual simulation felt real. This was evident when students mentioned that they were able to develop behaviors required for an optometry clinician in the virtual environment and that the simulation gave them *“The feel of assessing a real patient.”* (S32). Students’ identification of the importance of context, conceptual, functional and task fidelity suggest that the professional practice of optometry can be represented in virtual simulation to produce psychological fidelity by the consideration of these domains (Fig. 3).

In this study, these types of fidelity, subthemes, were reflected by the students as enablers of the development of cognitive and affective skills using virtual simulation in optometry education that are supported by the authenticity and realism of the virtual world. To generate a successful learning and assessment strategy in virtual simulation the design needs to also consider the level of fidelity required. To create a high-fidelity environment, developers need to achieve high physical fidelity in addition to high cognitive fidelity as keystones. Although high physical fidelity is often a goal in simulation development, achieving this can be costly in terms of resources. In addition, a higher level of detail increases the cognitive workload for students, and as a result can actually impede learning [32, 37]. In fact, in research on complex procedures in surgical training, for higher order cognitive tasks, simpler simulations can achieve elevated skills training [39]. Focusing on psychological fidelity is more cost effective than attempting to re-engineer the environment to achieve physical fidelity [10]. The secondary advantage is that lower fidelity computer simulations such as the VDECC can be used to host varied tasks without any degradation in cognitive or psychological fidelity, enabling greater contextual variety [37]. In this design the lack of physical interaction may have been an advantage because of the focus on the cognitive and affective skills in optometry and the development of these skills.

This study identified the dimensions of psychological fidelity within the VDCECC that students identified as important and that each contributed to support a sense of realism. This finding provides important insight into the features of virtual environments that enable the development of optometry students’ self-reported cognitive and affective skills and their professional identity. Further investigation into the impact that the degree of fidelity has on learning optometric skills needs to be undertaken.

### Virtual simulation as motivation in optometry education

The virtual simulation strategy using the VDCECC provided disciplinary context and professional relevance to the unit content, and this increased students’ motivation for learning. Students reported that they held preference for this learning strategy, and though it should not replace traditional lectures or in-person procedural skills tutorials, there is a place for it in future optometric education. The findings of this study demonstrate that virtual simulation in optometry education was effective in motivating first year pre-clinical optometry students to study unit content and also to research beyond the content delivered in the unit. Motivating first year students to engage in study beyond that which is assessed has always been challenging and these results encourage further efforts to explore and embed virtual simulation in optometric education.

### Core competency cognitive and affective skill development

The development of core cognitive and affective clinical skills is often not explicitly focused on during pre-clinical studies in the context of future professional roles. These skills are essential to clinical practice and outlined in graduate standards but are thought to be acquired later in their studies and refined once the student becomes a graduate optometrist, accrues experience in the workforce and participates in continual professional development [10]. In this study, students’ self-appraised their cognitive and affective skill development as improving through the virtual simulation experience and that this occurs through associating the meaning of information and development through interaction with equipment and the visceral experience of the virtual simulation activities. Future studies incorporating the use of a control group could provide evidence that the development of cognitive and affective skills could be supported earlier in optometric education through virtual simulation. If students can be provided with training and assessment tools that help them develop the affective skills and metacognitive process required to integrate such cognitive skills before their first placement in an optometry clinic, they may be less overwhelmed by early clinical practice and better able to assimilate the barrage of information they face in early clinical training. Studies on simulation-based education in nursing practice have reported that simulated learning experiences improve knowledge, communication, self-efficacy, motivation and competence but not critical thinking [21]. As there was a self-reported improvement in clinical reasoning in this study, future studies should investigate the ability of virtual simulation activities in optometry education to improve clinical reasoning skills.

### The influence of the learning environment in the VDCECC on optometry students’ self-reported learning

The influence of the learning environment in the VDCECC gave students an opportunity to apply learning to professional practice, develop their professional identity and appreciate their future roles and responsibilities. In designing the VDCECC simulation, the aim was to provide an authentic environment and experience to help students identify the relevance of their learning to the challenges they will face in their future professional practice. Participants’ comments demonstrated the powerful capacity of the simulation to inspire students to take on the responsibility of the clinician, *“starting from the moment they come into the clinic to making future plans felt like work experience”* (S34). In the virtual simulation, the framing, scaffolding and debriefing of simulation scenarios provide opportunities for these students to be a professional in an environment that reaffirms them through the fidelity and realism and also in how their decisions impacted their virtual client’s clinical outcomes. An outcome of this was that the student saw themselves performing in their future professional role which may result from students seeing their own work as an extension of themselves and why they started to think of themselves as “*in the shoes of an actual Optometrist”* (P30) [40]. Several students mentioned the similarity of this experience to what they expected would occur in future clinical placements, perhaps because students in optometric education are often learning skills that they will not currently use but will need in the future. By contrast in the VDCECC students immediately performed their developing skills with a (virtual) patient*.* This identifies that the VDCECC is perceived by students to add value to optometric education through the immediate application of learning in authentic contexts, and there may be scope to continue to evolve teaching practice to encompass virtual clinical placements through these methods.

### Limitations

This study investigates students’ self-reported skill development and did not objectively measure changes in students’ skills as a result of their experience in the virtual clinic. However, the findings of this study in relation to students’ self-reported skill development and motivation are important given the motivating impact that the perception of learning progress provides. The survey instrument was developed by a panel of experts for content and construct validity. Panel members were either qualified optometrists or experts in instructional design as no published instruments were available. As this was part of a quality assurance process of a novel teaching environment it was not formally validated and therefore further work is needed to confirm the generalizability of the results. We did not use a pre- and post-test as the reliability of the survey instrument had not been formally evaluated.

The rollout of the virtual clinic coincided with the COVID-19 pandemic spread, and students were not able to attend campus during their trimester of study. Students had experienced only 2 weeks on campus before moving to online study. This virtual environment was, for some, the only real tangible connection that they had with the physical university campus. It is possible therefore, that the virtual environment was more keenly appreciated by students at a time where face to face interaction was not possible.

## Conclusions

This study is the first to report on the perceived value for students of virtual simulation in optometric education. This study demonstrated that virtual simulation is motivating for students and increased their ambition to become an optometrist. Importantly, the study identified the dimensions of fidelity and aspects of realism in the virtual simulation that contributed to creating a virtual world, that supports pre-clinical students’ learning through being centred in the role as a clinician. In this learning environment students reported they were able to develop their professional identify and have an appreciation for their future roles. Students also reported through self-appraisal an increase in cognitive and affective skills and further studies are needed to identify the optimal level of fidelity required for skill development in virtual simulation in optometric education. Overall, this study showed that optometry students value learning in virtual simulation and it can play an important role in curriculum design for innovative learning strategies such as virtual placements.

## Supplementary Information

**Additional file 1.** Student Feedback Survey.

## Data Availability

The dataset generated and analyzed during the current study is available to the authors but is not publicly available due to ethical guidelines. The datasets used and/or analyzed during the current study available from the corresponding author on reasonable request.

## Abbreviations

DCECC: Deakin Collaborative Eye Care Clinic
VDCECC: Virtual Deakin Collaborative Eye Care Clinic
