# Supplementary material for: The perceived value and impact of virtual simulation-based education on students’ learning: a mixed methods study
Source: BMC Med Educ. 2022 Nov 30;22:823. doi: 10.1186/s12909-022-03912-8 (PMC9709374; doi:10.1186/s12909-022-03912-8)
Supplement: Supplementary file 1 — Additional file 1. Student Feedback Survey. [file 12909_2022_3912_MOESM1_ESM.pdf]

# HMO102 Science of Vision AT3 - Student Feedback Survey

In HMO102 you completed a virtual simulation assessment managing a patient case as a member of a team. This survey asks a series of questions designed to explore your experience and reflections on this task.

1. What gender are you?
    - Male
    - Female
    - Other
    - Prefer not to say

2. What is your age?

3. What qualifications do you currently have? E.g. Bachelor's degree in nursing

4. What is your previous experience in an optometry setting?

    - I have visited a practice to purchase glasses
    - I have had an eye test by an optometrist before
    - I have browsed an optometry store/retail store before
    - I have visited a university eye clinic before
    - I have worked in an optometry practice before as support staff
    - Other (Please describe)

**Please answer the following questions and where required indicate the extent to which you agree with statements by placing a cross in the corresponding box:**

5. Did you use the Virtual Deakin Collaborative Eye Care Clinic?
   If yes,
6. How and why did you use it?

7. How accurately does the Virtual Deakin Collaborate Eye Care Clinic represent your perception of an optometry clinic?

| Very inaccurate | Not accurate | Unsure | accurate | Very accurate |
|---|---|---|---|---|
| | | | | |

Please explain your answer

8. What benefits did your participation in the Virtual Deakin Collaborative Eye Care Clinic in Assessment Task 3 provide you?

9. Did you find any challenges or disadvantages in using the Virtual Deakin Collaborative Eye Care Clinic in Assessment Task 3?

10. What supported you to undertake Assessment Task 3?

11. Was there any other support you needed for this task?

12. I am comfortable using a computer to point and click

| Strongly disagree | Disagree | Unsure | Agree | Strongly agree |
|---|---|---|---|---|
| | | | | |

13. The virtual simulation of the Virtual Deakin Collaborative Eyecare Clinic for Assessment Task 3: was realistic

| Strongly disagree | Disagree | Unsure | Agree | Strongly agree |
|---|---|---|---|---|
| | | | | |

was relevant to my learning

| Strongly disagree | Disagree | Unsure | Agree | Strongly agree |
|---|---|---|---|---|
| | | | | |

motivated me to learn the unit's content so I could apply theory and evidence to a patient scenario

| Strongly disagree | Disagree | Unsure | Agree | Strongly agree |
|---|---|---|---|---|
| | | | | |

motivated me to research topics beyond the material provided such as online lectures and readings.

| Strongly disagree | Disagree | Unsure | Agree | Strongly agree |
|---|---|---|---|---|
| | | | | |

14. I would prefer virtual simulation instead of a regular lecture (e.g. PowerPoint presentation)

| Never | Less frequently | Sometimes | More frequently | Always |
|---|---|---|---|---|
| | | | | |

Please explain your answer

**This assessment involved online lectures, flipped classrooms, group application tasks, facilitated discussions by context experts and self-reflection. We are interested in hearing from you about how effective they were and why so we can continue to develop these activities in the Bachelor of Vision Science/Master of Optometry course.**

15. What skills did you begin developing through the virtual simulation experience?

16. How did the virtual simulation context of Assessment Task 3 improve your clinical reasoning skills?

| Reduced a great deal | Reduced | No change | Improved a little | Improved a great deal |
|---|---|---|---|---|
| | | | | |

What parts of Assessment Task 3 impacted this and why?

17. How did the virtual simulation context of Assessment Task 3 improve your knowledge of evidence-based practice?

| Reduced a great deal | Reduced | No change | Improved a little | Improved a great deal |
|---|---|---|---|---|
| | | | | |

What parts of Assessment Task 3 impacted this and why?

18. How did the virtual simulation context of Assessment Task 3 improve your clinical knowledge?

| Reduced a great deal | Reduced | No change | Improved a little | Improved a great deal |
|---|---|---|---|---|
| | | | | |

What parts of Assessment Task 3 impacted this and why?

19. How did the virtual simulation context of Assessment Task 3 improve your communication skills?

| Reduced a great deal | Reduced | No change | Improved a little | Improved a great deal |
|---|---|---|---|---|
| | | | | |

What parts of Assessment Task 3 impacted this and why?

20. How did the virtual simulation context of Assessment Task 3 improve your understanding of patient care skills?

| Reduced a great deal | Reduced | No change | Improved a little | Improved a great deal |
|---|---|---|---|---|

| | | | | |
|---|---|---|---|---|
| | | | | |

What parts of Assessment Task 3 impacted this and why?

21. How did the virtual simulation context of Assessment Task 3 improve your team work skills?

| Reduced a great deal | Reduced | No change | Improved a little | Improved a great deal |
|---|---|---|---|---|
| | | | | |

What parts of Assessment Task 3 impacted this and why?

22. After participating in the virtual simulation context of Assessment Task 3:
- the next time I encounter a real or simulated patient I will feel…

| Less confident | Unchanged confidence | Increased confidence |
|---|---|---|
| | | |

I have a good understanding of how I will behave as an optometrist

| Strongly disagree | Disagree | Unsure | Agree | Strongly agree |
|---|---|---|---|---|
| | | | | |

We understand people enroll in this course for a range of reasons and you may not be certain at this stage about your desire to become an optometrist, but please answer as honestly and openly as you can knowing this survey is anonymous.

23. Reflecting on your participation in the virtual simulation context of Assessment Task 3 what effect did it have on your ambition to become an optometrist?

| Strongly decreased | Decreased | Unsure | Increased | Strongly Increased |
|---|---|---|---|---|
| | | | | |

24. What aspects of Assessment Task 3 helped you understand what it is like to be an optometrist and what specific components helped you with this?

25. Do you believe virtual simulation in optometry education has the potential to impact the way you learn optometry? If so, how.
